# A Synthetic Biology Approach to Transgene Expression in Insects

**DOI:** 10.1021/acssynbio.4c00250

**Published:** 2024-08-28

**Authors:** Philip T. Leftwich, Jessica C. Purcell, Michelle A. E. Anderson, Rennos Fragkoudis, Sanjay Basu, Gareth Lycett, Luke Alphey

**Affiliations:** 1 Arthropod Genetics, The Pirbright Institute, Ash Road, Pirbright, GU24 0HN, U.K.; 2 Arbovirus Pathogenesis Group, The Pirbright Institute, Ash Road, Pirbright, GU24 0HN, U.K.; 3 University of Edinburgh, Edinburgh Genome Foundry, Centre for Mammalian Synthetic Biology, Michael Swann Building, Max Born Crescent, Edinburgh, EH9 3BF, U.K.; 4 Liverpool School of Tropical Medicine, Pembroke Place, Liverpool, L3 5QA, U.K.

**Keywords:** Promoter, mosquito, genetic engineering, insect, mRNA, translation

## Abstract

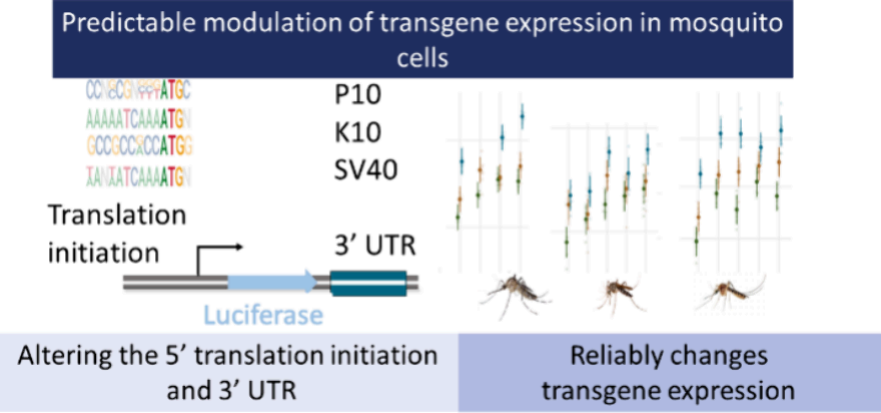

The ability to control gene expression is pivotal in genetic engineering and synthetic biology. However, in most nonmodel and pest insect species, empirical evidence for predictable modulation of gene expression levels is lacking. This knowledge gap is critical for genetic control systems, particularly in mosquitoes, where transgenic methods offer novel routes for pest control. Commonly, the choice of RNA polymerase II promoter (Pol II) is the primary method for controlling gene expression, but the options are limited. To address this, we developed a systematic approach to characterize modifications in translation initiation sequences (TIS) and 3′ untranslated regions (UTR) of transgenes, enabling the creation of a toolbox for gene expression modulation in mosquitoes and potentially other insects. The approach demonstrated highly predictable gene expression changes across various cell lines and 5′ regulatory sequences, representing a significant advancement in mosquito synthetic biology gene expression tools.

## Introduction

The ability to control the strength of expression of transgenes in a species of interest has underpinned genetic engineering and synthetic biology from their conception. There is, however, a lack of robust, empirical evidence for predictable modulation of gene expression levels in most nonmodel and pest insect species.^1,2^ In mosquitoes, transgenic methods afford novel routes for pest control, however, genetic control systems depend on precise gene expression, so this lack of information is a critical gap in our technical capability. Commonly the selection of RNA polymerase II promoter (Pol II) can dictate the amount, timing, and spatial specificity of gene transcription. A limited number of promoters of viral origin are sometimes used, as these are active across a range of insect species, however there are applications for characterizing endogenous promoters of varying activity. The choice of promoter is often determined by a requirement for specific spatial and/or temporal regulation, with few options for controlling expression level by this route beyond bespoke analysis of endogenous promoters.

Other methods commonly employed for modulating gene expression involve modifications to the mRNA sequences of the 5′ and 3′ untranslated regions (UTR), flanking the coding sequence of a transgene. Although these regions do not contribute to the final protein, they play crucial roles in mRNA stability and translation efficiency. In particular, the translation initiation sequence (TIS), a short (∼10nt) segment within the 5′ UTR just upstream of the start codon, has been shown to significantly impact translation efficiency in both vertebrates and invertebrates.^3−6^ By using different TIS sequences, predictable changes in transgene expression can be achieved.^2,6,7^

The 3′UTR is more closely associated with mediating the termination of translation and ensuring efficient recycling of the translation complex, enabling multiple translations from the same mRNA molecule.^8,9^ In insect transgenesis, exogenous 3′UTR sequences are routinely used, including the viral-derived simian virus 40 (SV40) 3′UTR,^11^ the P10 baculovirus 3′ UTR from *Autographa californica nucleopolyhedrovirus* (AcNPV)^12^ and the 3′UTR of the *K10* gene from *Drosophila melanogaster*.^13^

Despite the widespread use of these 3′UTR sequences, their relative efficacies are often based on anecdotal evidence. Strategically manipulating these untranslated regions provides a valuable approach to finely tune and optimize transgene expression in insect transgenesis.

We developed a systematic approach to characterizing TIS and 3′UTR modifications to transgenes to build a toolbox for modulating gene expression in mosquitoes, and potentially other insects, when combined with viral or endogenous culicine 5′ regulatory DNA sequences–hereafter referred to as promoters (though strictly these DNA regions may also contain enhancers, silencers, or binding sites for transcription factors required for proper regulation of gene expression). This toolbox provides an efficient way of expanding available Pol II promoters and affords routes to generating better regulation of activity of promoters across the species barrier. We initially tested the activities of the viral promoter HR5-IE1^10^ with a fully factorial combination of TIS and 3′UTR sequences in *Aedes aegypti, Aedes albopictus, Culex quinquefasciatus* and *Spodoptera frugiperda* cell lines. We then developed this further by taking the TIS/3′UTR combinations that produced the highest, lowest and median expression levels and demonstrated the highly replicable gene expression modulation effects in a panel of promoters.

We found that gene expression changes are highly predictable across a wide range of cell lines and promoter sequences. In conjunction with the characterization of several endogenous culicine promoters, this represents a significant advance in the available gene expression tools for mosquito synthetic biology.

## Results and Discussion

We first measured the activity of five translation initiation sequences (TIS) (Table S1; BmLo, Syn21, Kozak, BmHi, and Lep)^1−3,15^ and three different 3′UTRs (K10, SV40, P10)^11−13^ in a fully factorial design all downstream of the HR5-IE1 promoter.^14^ In total, we produced 15 different constructs and tested these in five different insect cell lines, from three disease relevant Culicine mosquito species (*A. aegypti (Aag2)*, *A. albopictus (U4.4* and *C6.36)* and *C. quinquefasciatus (Hsu)*) and one Lepidopteran species (*S. frugiperda* (*Sf9*)).

**Figure 1:**
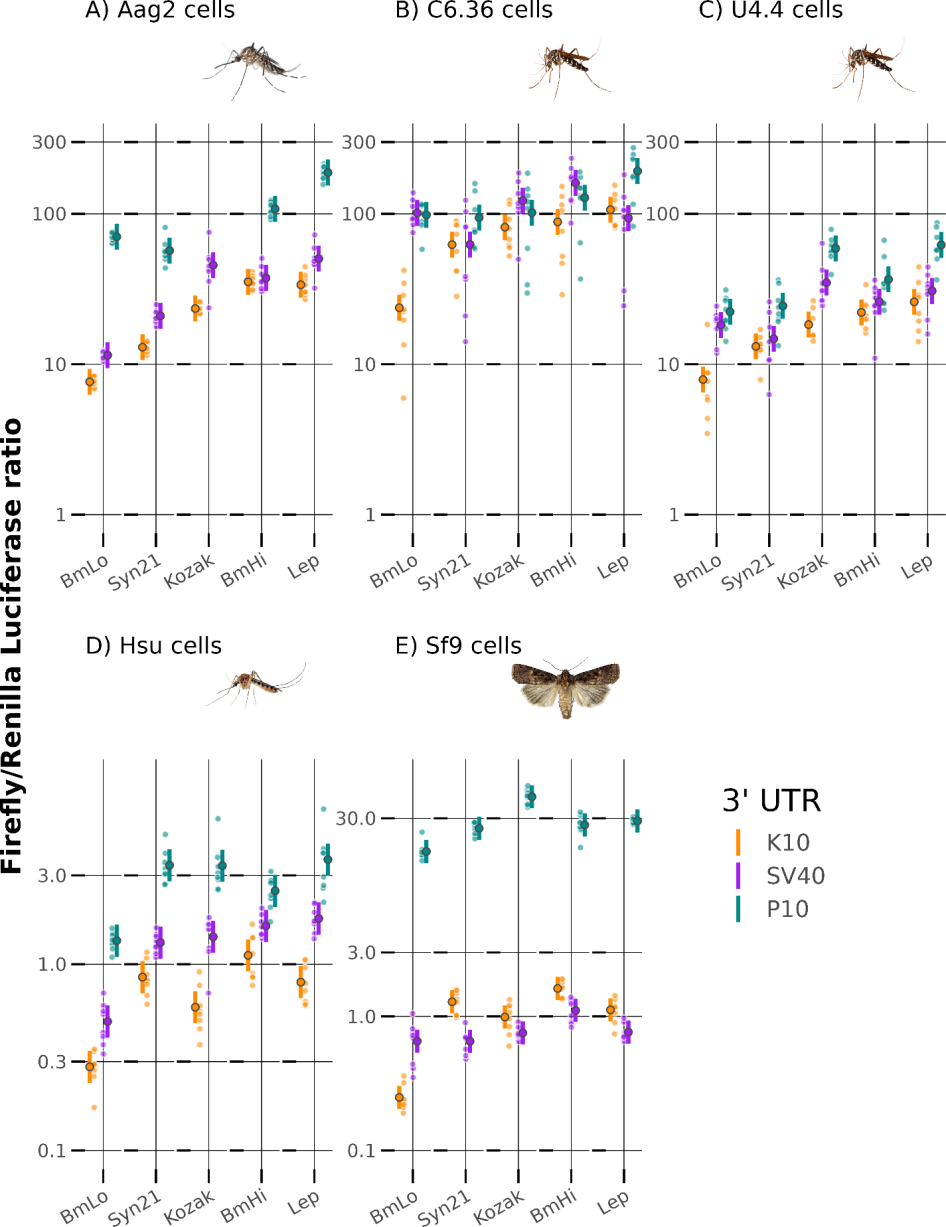
Combinations of translation initiation sequences (TIS) and 3′UTRs produce highly replicable gene expression across a range of insect cell lines. Ratios of FF luciferase compared to a RL control were used to measure activity; UTRs are organized left to right by average relative activity and nested within TIS also organized left to right by average relative activity. Large symbols and error bars represent mean and associated approximate 95% confidence intervals estimated with a generalized linear mixed model with a Gamma error distribution, raw data is shown as small points.

We found a highly replicable pattern of gene expression modulation across all five tested cell lines (Figure 1). Averaged across cell lines, the choice of TIS could produce up to a 2.55 relative-fold change in luciferase expression (95% CI 2.28–2.84; Table S2), while the choice of 3′UTR produced up to a 4.88 relative-fold change in luciferase expression (95% CI 4.52–5.26; Table S2).

The estimates from our analysis indicate that TIS sequences are mainly insensitive to cell type and behave remarkably consistently (F_16,518_ = 11, *P* < 0.001; Table S3). By contrast, the effect of the 3′UTR sequence on transgene expression was much more strongly affected by cell type (F_8,518_ = 250, *P* < 0.001; Table S3). Expression from constructs with P10 had notably higher expression than expected in Sf9 cells and much lower in both C6/36 and U4.4 cells. Significant interactions between TIS sequences and 3′UTR sequences were small (F_8,518_ = 20, *P* < 0.001; Table S3) therefore transcriptional activity appears to be primarily an additive effect when pairing TIS and 3′UTR sequences. This makes the “plug and play” notion of pairing different synthetic components together highly attractive, as effects on transgene expression are highly predictable.

The TIS/3′UTR combinations with the lowest (BmLo-K10) and highest (Lep-P10) expressions (18.2 (95% CI 13–22) relative-fold expression difference); were the same across all cell lines (Figure 1). To generalize our results, we decided to expand the range of promoter sequences tested by taking the BmLo-K10, Lep-P10 and Kozak-SV40 combinations and testing them with additional promoter sequences. In total, we tested seven regulatory sequences from four endogenous promoters from Culicine mosquitoes: two variants of the *hsp83* promoter^16^ (1.4kb and 888bp) (AAEL011708), from *A. aegypti* with the large (c.4.2kb) intronic sequence of the 5′UTR truncated to retain minimal acceptor and donor regions, three engineered variants of the *Polyubiquitin* promoter (AAEL003888) from *A. aegypti*,^17^ along with *Polyubiquitin* from *C. quinquefasciatus* (CPIJ010919) and we demonstrate the first use case for a new endogenous promoter *A. albopictus* derived *Polyubiquitin* (AALF002118). These, along with OpIE2 were tested in Aag2 and U4.4 cells.

**Figure 2:**
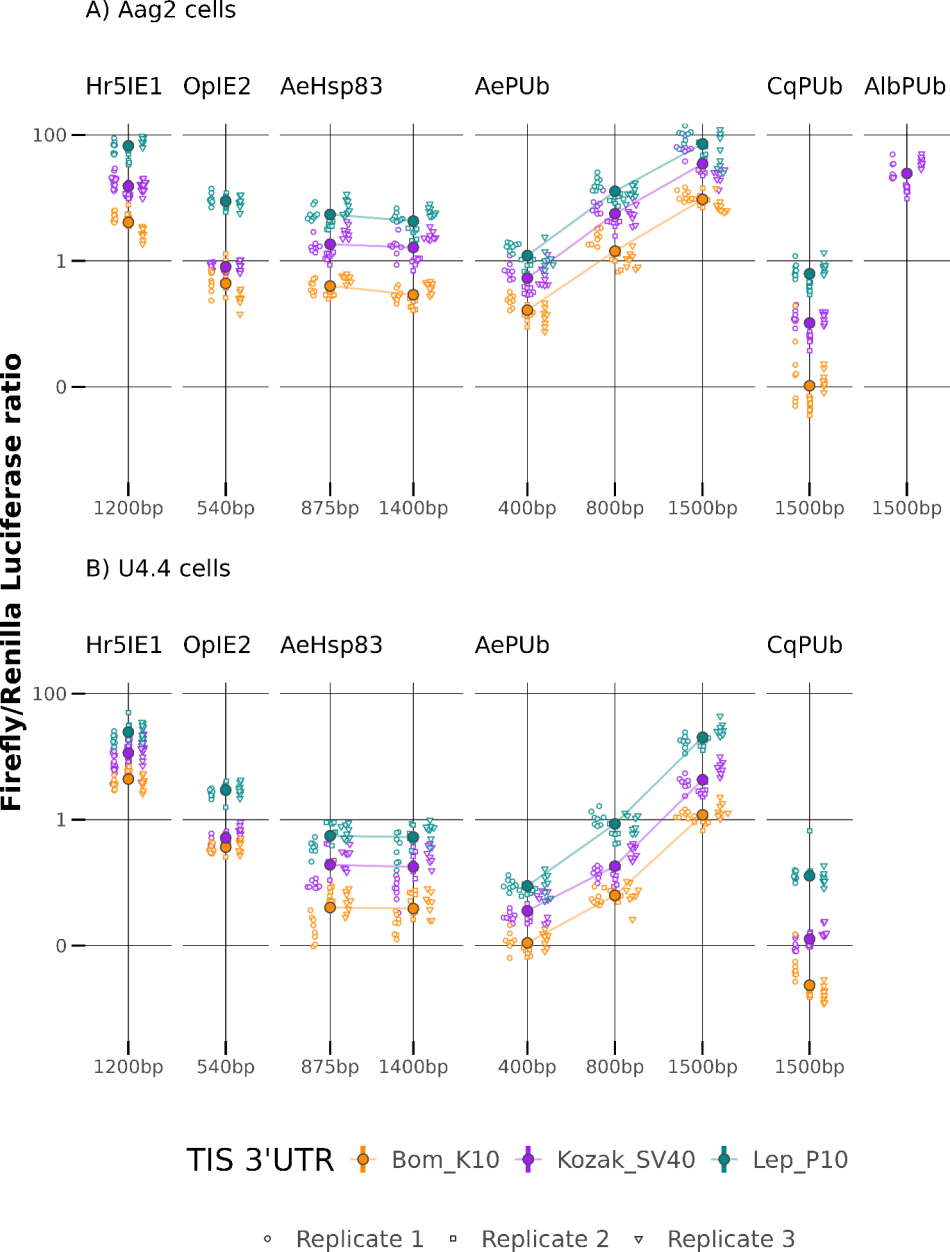
Fine-scale modulation of transgene expression with TIS/3′UTR combinations is highly replicable across a range of synthetic and endogenous promoters in two Culicine mosquito cell lines: (A) Aag2 (*A. aegypti*) cells and (B) *A. albopictus*-derived U4.4 cells. Ratios of FF luciferase compared to a RL control were used to measure activity; TIS/3′UTR combinations are organized left to right by average relative activity, with the *x*-axis indicating the size of promoter fragments in base pairs (bp). Large solid symbols and error bars represent mean and associated approximate 95% confidence intervals estimated with a generalized linear mixed model with a Gamma error distribution; raw data is shown as open symbols. Lines connect promoters of the same origin.

As expected, promoters of viral origin behaved very consistently across both cell lines, while endogenous promoters responded in a more cell-specific manner (Figure 2, Table S4). The *hsp83* promoter sequences produced equivalent levels of gene expression to OpIE2 when in Aag2 cells, but this was lower in U4.4 cells. Shortening this sequence by removing c.500bp upstream produced no significant reduction in gene expression. Polyubiquitin-derived promoter sequences generally produced the highest levels of gene expression; fluctuations in the strength of expression across cell lines may reflect the evolutionary origins of each sequence (*C. quinquefasciatus* derived sequence displayed lower activity than *A. albopictus* for example).

The effects of TIS/3′UTR modification on gene expression were remarkably consistent for all promoter sequences. We found minimal differences in the responses between different regulatory sequences and TIS/3′UTR combinations (Promoter: F_8,1207_ = 354.91, *P* < 0.001; TIS/3′UTR: F_2,1207_ = 509.96, *P* < 0.001; Interaction effect: F_14,1193_ = 7.26, *P* < 0.001; Figure 2, Table S5), indicating that these act largely independently.

We have developed a straightforward method for modulating transgene expression in Culicine mosquitoes using a combinatorial approach that enables fine-scale manipulation. Our experiments demonstrated that TIS and 3′UTR sequences consistently produce highly predictable outcomes on transgene expression irrespective of promoter sequence or cell line. While this work was conducted in cultured cell lines, previous research strongly suggests that these findings will effectively translate to whole-organism transgene expression.^1,17^ We are confident that this will be a a valuable resource for researchers in synthetic biology, genetic modification, and mosquito genetic control.

## Methods

### Plasmids, Cells, Transfections, and Luciferase Assay

Cells were seeded in 96-well plates 1 day before transfection with TransIT-PRO transfection kit (Mirus Bio, Madison, WI, US) according to manufacturer’s recommendations. Master mixes were prepared for eight wells of a 96-well plate, as replicate wells per experimental construct. This was repeated in three replicate experiments. Per well, transfection amounts are listed for each cell line in Supporting Information. Complete plasmid sequences are currently available as genbank files on Github (see below) and will be available through NCBI upon publication.

Two days after transfection, cells were washed twice with ion-free PBS, lysed with 1× Passive Lysis Buffer then analyzed using the Dual-Luciferase Assay kit on a GloMax multi+ plate reader (Promega, Southampton, UK).

General cell maintenance and plasmid information is described in Supporting Information (Tables S6–S9).

## Data Availability

Scripts and raw data can be found at Github (https://github.com/Philip-Leftwich/Pol2-promoters). Complete information on analyses can be found in Supporting Information.

## Supporting Information Available

Table S1, model summaries for experiment one; Table S2, pairwise contrasts for experiment one; Table S3, ANOVA tables for experiment one; Table S4, model summaries for experiment two; Table S5, ANOVA tables for experiment two; Table S6, transfection amounts per cell line; Table S7, lysate volumes per experiment; Table S8, DNA sequences; Table S9, brief descriptors of plasmids; additional methods for cell culture, transfections, dual luciferase assays DNA sequences, and references (PDF)
