# Supplementary material for: A Synthetic Biology Approach to Transgene Expression in Insects
Source: ACS Synth Biol. 2024 Aug 28;13(9):3041–5. doi: 10.1021/acssynbio.4c00250 (PMC11421086; doi:10.1021/acssynbio.4c00250)
Supplement: Supplementary file 1 — sb4c00250_si_001.pdf [file sb4c00250_si_001.pdf]

Supplementary Methods

A synthetic biology approach to transgene expression in insects

Philip T. Leftwich[1,2]±, Jessica C. Purcell[1]±, Michelle A.E. Anderson[1,3], Rennos Fragkoudis[4,5], Sanjay Basu[1], Gareth Lycett[6], Luke Alphey[1,3]

[1]Arthropod Genetics, The Pirbright Institute, Ash Road, Pirbright, GU24 0HN, U.K.

[2] Current address: School of Biological Sciences, University of East Anglia, Norwich, NR4 7TJ, U.K.

[3] Current address: Department of Biology, University of York, Heslington, YO10 5DD, U.K.

[4]Arbovirus Pathogenesis Group, The Pirbright Institute, Ash Road, Pirbright, GU24 0HN, U.K.

[5] University of Edinburgh, Edinburgh Genome Foundry , Centre for Mammalian Synthetic Biology, Michael Swann Building , Max Born Crescent , Edinburgh, EH9 3BF

[6] Liverpool School of Tropical Medicine , Pembroke Place, Liverpool , L3 5QA, UK

± Joint first authors

Email: p.leftwich@uea.ac.uk, luke.alphey@york.ac.uk

Contents

Contents 1

1. Analysis 3

2 Experiment One 4

Table S1. 4

Table S2.. 10

Table S3. 11

3 Experiment Two 12

Table S4. 12

Table S5.. 22

4. Methods 23

Table S6. 23

4.1 Dual luciferase assay....23

Figure S1. 24

Table S7. 24

5. DNA sequences....25

Table S8.. 25

Table S9. 31

References....33

1. Analysis

We carried out all analyses in R version 4.3.1 (R Development Core Team)

Luciferase readings were normalized for transfection by dividing the firefly activity by the Renilla activity. Data were analyzed by generalized linear models using a gamma error distribution with a log link, we included promoter, translation initation sequence, 3'UTR and cell line as factorial explanatory variables with all possible interactions. There is minor overdispersion because of heterogeneity among cell types, likely caused by the different basal expression levels of the OpIE2 promoter expressing Renilla luciferase. After each model was fitted, we used the 'emmeans' package (Lengh 2023) to back transform regression coefficients and calculate the estimated normalised mean expression with approximate 95% confidence intervals for each construct.

2 Experiment One

Table S1. Fixed effects table for the generalized linear model (GLMM) fitted to the Luciferase Ratio detected in the engineered plasmids in experiment one – testing Hr5IE1 promoter with a combination of TIS, 3'UTR and cell types

	**Value**		
Predictors	*Estimates*	*CI*	*p*
(Intercept)	35.34	29.18 – 43.36	**<0.001**
Context [BmLo]	0.22	0.16 – 0.29	**<0.001**
Context [Kozak]	0.66	0.50 – 0.88	**0.004**
Context [Lep]	0.95	0.72 – 1.26	0.745
Context [Syn21]	0.37	0.28 – 0.49	**<0.001**
UTR [P10]	3.05	2.30 – 4.03	**<0.001**
UTR [SV40]	1.06	0.80 – 1.40	0.695
Cell Line [C6.36..Ae..albopictus.]	2.50	1.89 – 3.31	**<0.001**
Cell Line [Hsu..Cu..quinquefasciatus.]	0.03	0.02 – 0.04	**<0.001**
Cell Line [Sf9..S..frugiperda.]	0.05	0.03 – 0.06	**<0.001**
Cell Line [U4.4..Ae..albopictus.]	0.63	0.47 – 0.83	**0.001**
Context [BmLo] × UTR [P10]	3.02	2.03 – 4.49	**<0.001**
Context [Kozak] × UTR [P10]	1.94	1.30 – 2.88	**0.001**
Context [Lep] × UTR [P10]	1.83	1.23 – 2.72	**0.003**
Context [Syn21] × UTR [P10]	1.44	0.97 – 2.14	0.071
Context [BmLo] × UTR [SV40]	1.42	0.96 – 2.11	0.082

Context [Kozak] × UTR [SV40]	1.84	1.24 – 2.73	**0.003**
Context [Lep] × UTR [SV40]	1.41	0.95 – 2.10	0.089
Context [Syn21] × UTR [SV40]	1.53	1.03 – 2.27	**0.036**
Context [BmLo] × Cell Line [C6.36..Ae..albopictus.]	1.24	0.84 – 1.85	0.283
Context [Kozak] × Cell Line [C6.36..Ae..albopictus.]	1.39	0.94 – 2.07	0.102
Context [Lep] × Cell Line [C6.36..Ae..albopictus.]	1.26	0.85 – 1.87	0.250
Context [Syn21] × Cell Line [C6.36..Ae..albopictus.]	1.92	1.29 – 2.86	**0.001**
Context [BmLo] × Cell Line [Hsu..Cu..quinquefasciatus.]	1.17	0.79 – 1.73	0.445
Context [Kozak] × Cell Line [Hsu..Cu..quinquefasciatus.]	0.79	0.53 – 1.18	0.255
Context [Lep] × Cell Line [Hsu..Cu..quinquefasciatus.]	0.75	0.51 – 1.12	0.160
Context [Syn21] × Cell Line [Hsu..Cu..quinquefasciatus.]	2.08	1.40 – 3.10	**<0.001**
Context [BmLo] × Cell Line [Sf9..S..frugiperda.]	0.71	0.48 – 1.06	0.093
Context [Kozak] × Cell Line [Sf9..S..frugiperda.]	0.92	0.62 – 1.37	0.689
Context [Lep] × Cell Line [Sf9..S..frugiperda.]	0.73	0.49 – 1.08	0.112

TIS [Syn21] × Cell Line [Sf9..S..frugiperda.]	2.17	1.46 – 3.22	**<0.001**
TIS [BmLo] × Cell Line [U4.4..Ae..albopictus.]	1.65	1.11 – 2.46	**0.013**
TIS [Kozak] × Cell Line [U4.4..Ae..albopictus.]	1.25	0.84 – 1.86	0.267
TIS [Lep] × Cell Line [U4.4..Ae..albopictus.]	1.23	0.83 – 1.83	0.304
TIS [Syn21] × Cell Line [U4.4..Ae..albopictus.]	1.62	1.09 – 2.40	**0.017**
UTR [P10] × Cell Line [C6.36..Ae..albopictus.]	0.47	0.32 – 0.71	**<0.001**
UTR [SV40] × Cell Line [C6.36..Ae..albopictus.]	1.72	1.15 – 2.55	**0.007**
UTR [P10] × Cell Line [Hsu..Cu..quinquefasciatus.]	0.73	0.49 – 1.08	0.117
UTR [SV40] × Cell Line [Hsu..Cu..quinquefasciatus.]	1.36	0.92 – 2.02	0.127
UTR [P10] × Cell Line [Sf9..S..frugiperda.]	5.43	3.66 – 8.07	**<0.001**
UTR [SV40] × Cell Line [Sf9..S..frugiperda.]	0.65	0.44 – 0.96	**0.032**
UTR [P10] × Cell Line [U4.4..Ae..albopictus.]	0.54	0.37 – 0.81	**0.003**
UTR [SV40] × Cell Line [U4.4..Ae..albopictus.]	1.11	0.75 – 1.65	0.596
(TIS [BmLo] × UTR [P10]) × Cell Line [C6.36..Ae..albopictus.]	0.95	0.54 – 1.66	0.850
(TIS [Kozak] × UTR [P10]) × Cell Line [C6.36..Ae..albopictus.]	0.45	0.25 – 0.78	**0.005**

(TIS [Lep] × UTR [P10]) × Cell Line [C6.36..Ae..albopictus.]	0.68	0.39 – 1.20	0.181
(TIS [Syn21] × UTR [P10]) × Cell Line [C6.36..Ae..albopictus.]	0.73	0.41 – 1.27	0.262
(TIS [BmLo] × UTR [SV40]) × Cell Line [C6.36..Ae..albopictus.]	1.65	0.94 – 2.90	0.078
(TIS [Kozak] × UTR [SV40]) × Cell Line [C6.36..Ae..albopictus.]	0.45	0.26 – 0.79	**0.005**
(TIS [Lep] × UTR [SV40]) × Cell Line [C6.36..Ae..albopictus.]	0.34	0.20 – 0.60	**<0.001**
(TIS [Syn21] × UTR [SV40]) × Cell Line [C6.36..Ae..albopictus.]	0.36	0.21 – 0.63	**<0.001**
(TIS [BmLo] × UTR [P10]) × Cell Line [Hsu..Cu..quinquefasciatus.]	0.71	0.41 – 1.24	0.230
(TIS [Kozak] × UTR [P10]) × Cell Line [Hsu..Cu..quinquefasciatus.]	1.34	0.76 – 2.34	0.309
(TIS [Lep] × UTR [P10]) × Cell Line [Hsu..Cu..quinquefasciatus.]	1.12	0.64 – 1.96	0.694
(TIS [Syn21] × UTR [P10]) × Cell Line [Hsu..Cu..quinquefasciatus.]	1.25	0.71 – 2.18	0.441
(TIS [BmLo] × UTR [SV40]) × Cell Line [Hsu..Cu..quinquefasciatus.]	0.86	0.49 – 1.50	0.587
(TIS [Kozak] × UTR [SV40]) × Cell Line [Hsu..Cu..quinquefasciatus.]	0.90	0.52 – 1.58	0.723
(TIS [Lep] × UTR [SV40]) × Cell Line [Hsu..Cu..quinquefasciatus.]	1.08	0.62 – 1.89	0.784

(TIS [Syn21] × UTR [SV40]) × Cell Line [Hsu..Cu..quinquefasciatus.]	0.70	0.40 – 1.22	0.207
(TIS [BmLo] × UTR [P10]) × Cell Line [Sf9..S..frugiperda.]	1.36	0.78 – 2.39	0.277
(TIS [Kozak] × UTR [P10]) × Cell Line [Sf9..S..frugiperda.]	1.37	0.78 – 2.40	0.270
(TIS [Lep] × UTR [P10]) × Cell Line [Sf9..S..frugiperda.]	0.85	0.48 – 1.48	0.558
(TIS [Syn21] × UTR [P10]) × Cell Line [Sf9..S..frugiperda.]	0.82	0.47 – 1.44	0.498
(TIS [BmLo] × UTR [SV40]) × Cell Line [Sf9..S..frugiperda.]	2.70	1.54 – 4.72	**0.001**
(TIS [Kozak] × UTR [SV40]) × Cell Line [Sf9..S..frugiperda.]	0.60	0.35 – 1.06	0.078
(TIS [Lep] × UTR [SV40]) × Cell Line [Sf9..S..frugiperda.]	0.71	0.40 – 1.24	0.223
(TIS [Syn21] × UTR [SV40]) × Cell Line [Sf9..S..frugiperda.]	0.49	0.28 – 0.85	**0.011**
(TIS [BmLo] × UTR [P10]) × Cell Line [U4.4..Ae..albopictus.]	0.56	0.32 – 0.99	**0.045**
(TIS [Lep] × UTR [P10]) × Cell Line [U4.4..Ae..albopictus.]	0.79	0.45 – 1.38	0.404
(TIS [Syn21] × UTR [P10]) × Cell Line [U4.4..Ae..albopictus.]	0.78	0.45 – 1.37	0.384
(TIS [BmLo] × UTR [SV40]) × Cell Line [U4.4..Ae..albopictus.]	1.38	0.79 – 2.42	0.260

(TIS [Kozak] × UTR [SV40]) × Cell Line [U4.4..Ae..albopictus.]	0.88	0.50 – 1.54	0.652
(TIS [Lep] × UTR [SV40]) × Cell Line [U4.4..Ae..albopictus.]	0.71	0.41 – 1.25	0.236
(TIS [Syn21] × UTR [SV40]) × Cell Line [U4.4..Ae..albopictus.]	0.63	0.36 – 1.10	0.101
Observations	592		
R^2 Nagelkerke	0.992		

Table S2. Pairwise contrasts table calculated from coefficients in full model (Table S1), back-calculated into a ratio difference on the original response scale.

Contrast	Mean Relative Expression	±95% CI
K10 / P10	0.20	0.19-0.22
K10 / SV40	0.71	0.66-0.76
P10 / SV40	3.47	3.22-3.73

Contrast	Mean Relative Expression	±95% CI
BmHi / BmLo	2.35	2.11-2.62
BmHi / Kozak	1.04	0.93-1.16
BmHi / Lep	0.92	0.83-1.03
BmHi / Syn21	1.52	1.36-1.69
BmLo / Kozak	0.44	0.39-0.49
BmLo / Lep	0.39	0.35-0.44
BmLo / Syn21	0.65	0.58-0.72
Kozak / Lep	0.89	0.79-0.99
Kozak / Syn21	1.46	1.31-1.64
Lep / Syn21	1.65	1.48-1.84

Table S3. ANOVA table F values calculated from stepwise removal of coefficients from full model (Table S1).

Term	Df	F value	Pr(>F)
TIS	4	36.43	< 0.001
UTR	2	299.72	< 0.001
Residuals	581		
Cell_Line	4	594.57	< 0.001
TIS:UTR	8	15.9	< 0.001
TIS:Cell_Line	16	8.57	< 0.001
UTR:Cell_Line	8	194.57	< 0.001
Residuals	549		
TIS:UTR:Cell_Line	31	4.2	< 0.001
Residuals	518		

3 Experiment Two

Table S4. Fixed effects table for the generalized linear model (GLMM) fitted to the Luciferase Ratio detected in the engineered plasmids in experiment two– testing a range of promoters with a fixed combination of Contexts (TIS, 3'UTR) and two cell types

	**Value**		
Predictors	*Estimates*	*CI*	*p*
(Intercept)	5.05	4.21 – 6.13	**<0.001**
Promoter [OpIE2]	0.10	0.07 – 0.13	**<0.001**
Promoter [shortHsp83]	0.08	0.06 – 0.10	**<0.001**
Promoter [Hsp83]	0.06	0.04 – 0.08	**<0.001**
Promoter [CqPUb]	0.00	0.00 – 0.00	**<0.001**
Promoter [400AePUb]	0.05	0.04 – 0.06	**<0.001**
Promoter [800AePUb]	0.47	0.36 – 0.62	**<0.001**
Promoter [AePUb]	2.19	1.68 – 2.87	**<0.001**
Promoter [AlbPUb]	1.49	1.18 – 1.88	**0.001**
Context [Kozak_SV40]	3.76	2.97 – 4.72	**<0.001**
Context [Lep_P10]	12.98	9.94 – 16.96	**<0.001**
Rep [MA34]	1.00	0.76 – 1.30	0.976
Rep [MA37]	0.54	0.42 – 0.71	**<0.001**
Cell Line [U4]	0.73	0.56 – 0.96	**0.023**
Promoter [OpIE2] × Context [Kozak_SV40]	0.47	0.33 – 0.68	**<0.001**
Promoter [shortHsp83] × Context [Kozak_SV40]	1.17	0.83 – 1.67	0.373
Promoter [Hsp83] × Context [Kozak_SV40]	1.59	1.12 – 2.26	**0.010**

Promoter [CqPUb] × Context [Kozak_SV40]	2.32	1.55 – 3.45	**<0.001**
Promoter [400AePUb] × Context [Kozak_SV40]	0.82	0.57 – 1.16	0.262
Promoter [800AePUb] × Context [Kozak_SV40]	0.85	0.60 – 1.22	0.378
Promoter [AePUb] × Context [Kozak_SV40]	1.49	1.05 – 2.13	**0.026**
Promoter [OpIE2] × Context [Lep_P10]	1.55	1.06 – 2.26	**0.023**
Promoter [shortHsp83] × Context [Lep_P10]	1.12	0.77 – 1.64	0.548
Promoter [Hsp83] × Context [Lep_P10]	1.30	0.89 – 1.89	0.176
Promoter [CqPUb] × Context [Lep_P10]	3.79	2.47 – 5.76	**<0.001**
Promoter [400AePUb] × Context [Lep_P10]	0.55	0.38 – 0.81	**0.002**
Promoter [800AePUb] × Context [Lep_P10]	0.56	0.39 – 0.82	**0.003**
Promoter [AePUb] × Context [Lep_P10]	0.68	0.47 – 0.99	**0.044**
Promoter [OpIE2] × Rep [MA34]	1.39	0.95 – 2.03	0.087
Promoter [shortHsp83] × Rep [MA34]	0.75	0.52 – 1.10	0.138
Promoter [Hsp83] × Rep [MA34]	0.72	0.50 – 1.06	0.093
Promoter [CqPUb] × Rep [MA34]	0.36	0.23 – 0.54	**<0.001**
Promoter [400AePUb] × Rep [MA34]	0.60	0.41 – 0.88	**0.008**
Promoter [800AePUb] × Rep [MA34]	0.48	0.33 – 0.69	**<0.001**

Promoter [AePUb] × Rep [MA34]	0.88	0.60 – 1.28	0.503
Promoter [AlbPUb] × Rep [MA34]	0.75	0.54 – 1.04	0.080
Promoter [OpIE2] × Rep [MA37]	0.98	0.67 – 1.43	0.906
Promoter [shortHsp83] × Rep [MA37]	2.47	1.69 – 3.60	**<0.001**
Promoter [Hsp83] × Rep [MA37]	2.42	1.66 – 3.53	**<0.001**
Promoter [CqPUb] × Rep [MA37]	1.69	1.11 – 2.57	**0.014**
Promoter [400AePUb] × Rep [MA37]	1.05	0.72 – 1.53	0.796
Promoter [800AePUb] × Rep [MA37]	0.84	0.58 – 1.23	0.367
Promoter [AePUb] × Rep [MA37]	1.31	0.90 – 1.92	0.157
Promoter [AlbPUb] × Rep [MA37]	1.64	1.19 – 2.28	**0.003**
Context [Kozak_SV40] × Rep [MA34]	0.65	0.47 – 0.91	**0.011**
Context [Lep_P10] × Rep [MA34]	0.78	0.54 – 1.14	0.206
Context [Kozak_SV40] × Rep [MA37]	1.51	1.09 – 2.09	**0.014**
Context [Lep_P10] × Rep [MA37]	2.54	1.74 – 3.70	**<0.001**
Promoter [OpIE2] × Cell Line [U4]	0.98	0.67 – 1.44	0.935
Promoter [shortHsp83] × Cell Line [U4]	0.07	0.05 – 0.10	**<0.001**
Promoter [Hsp83] × Cell Line [U4]	0.10	0.07 – 0.15	**<0.001**

Promoter [CqPUb] × Cell Line [U4]	0.36	0.23 – 0.55	**<0.001**
Promoter [400AePUb] × Cell Line [U4]	0.07	0.05 – 0.10	**<0.001**
Promoter [800AePUb] × Cell Line [U4]	0.03	0.02 – 0.05	**<0.001**
Promoter [AePUb] × Cell Line [U4]	0.15	0.10 – 0.22	**<0.001**
Context [Kozak_SV40] × Cell Line [U4]	0.61	0.44 – 0.84	**0.003**
Context [Lep_P10] × Cell Line [U4]	0.40	0.27 – 0.58	**<0.001**
Rep [MA34] × Cell Line [U4]	1.60	1.10 – 2.34	**0.014**
Rep [MA37] × Cell Line [U4]	1.96	1.34 – 2.85	**<0.001**
(Promoter [OpIE2] × Context [Kozak_SV40]) × Rep [MA34]	0.93	0.56 – 1.53	0.771
(Promoter [shortHsp83] × Context [Kozak_SV40]) × Rep [MA34]	1.38	0.83 – 2.27	0.211
(Promoter [Hsp83] × Context [Kozak_SV40]) × Rep [MA34]	1.18	0.72 – 1.94	0.517
(Promoter [CqPUb] × Context [Kozak_SV40]) × Rep [MA34]	2.11	1.24 – 3.61	**0.006**
(Promoter [400AePUb] × Context [Kozak_SV40]) × Rep [MA34]	1.22	0.74 – 2.01	0.437
(Promoter [800AePUb] × Context [Kozak_SV40]) × Rep [MA34]	1.66	1.00 – 2.73	**0.048**
(Promoter [AePUb] × Context [Kozak_SV40]) × Rep [MA34]	0.83	0.51 – 1.38	0.478

(Promoter [OpIE2] × Context [Lep_P10]) × Rep [MA34]	0.78	0.46 – 1.33	0.362
(Promoter [shortHsp83] × Context [Lep_P10]) × Rep [MA34]	1.09	0.64 – 1.86	0.744
(Promoter [Hsp83] × Context [Lep_P10]) × Rep [MA34]	0.91	0.53 – 1.55	0.729
(Promoter [CqPUb] × Context [Lep_P10]) × Rep [MA34]	1.88	1.07 – 3.32	**0.029**
(Promoter [400AePUb] × Context [Lep_P10]) × Rep [MA34]	1.14	0.67 – 1.94	0.632
(Promoter [800AePUb] × Context [Lep_P10]) × Rep [MA34]	1.46	0.86 – 2.50	0.161
(Promoter [AePUb] × Context [Lep_P10]) × Rep [MA34]	0.92	0.54 – 1.56	0.748
(Promoter [OpIE2] × Context [Kozak_SV40]) × Rep [MA37]	1.21	0.73 – 1.99	0.459
(Promoter [shortHsp83] × Context [Kozak_SV40]) × Rep [MA37]	0.84	0.51 – 1.38	0.493
(Promoter [Hsp83] × Context [Kozak_SV40]) × Rep [MA37]	0.73	0.44 – 1.20	0.211
(Promoter [CqPUb] × Context [Kozak_SV40]) × Rep [MA37]	0.72	0.42 – 1.23	0.223
(Promoter [400AePUb] × Context [Kozak_SV40]) × Rep [MA37]	0.96	0.59 – 1.59	0.888
(Promoter [800AePUb] × Context [Kozak_SV40]) × Rep [MA37]	1.12	0.68 – 1.85	0.655

(Promoter [AePUb] × Context [Kozak_SV40]) × Rep [MA37]	0.34	0.21 – 0.56	**<0.001**
(Promoter [OpIE2] × Context [Lep_P10]) × Rep [MA37]	0.65	0.38 – 1.11	0.113
(Promoter [shortHsp83] × Context [Lep_P10]) × Rep [MA37]	0.38	0.22 – 0.65	**<0.001**
(Promoter [Hsp83] × Context [Lep_P10]) × Rep [MA37]	0.37	0.22 – 0.63	**<0.001**
(Promoter [CqPUb] × Context [Lep_P10]) × Rep [MA37]	0.49	0.28 – 0.86	**0.012**
(Promoter [400AePUb] × Context [Lep_P10]) × Rep [MA37]	0.48	0.28 – 0.81	**0.006**
(Promoter [800AePUb] × Context [Lep_P10]) × Rep [MA37]	0.62	0.36 – 1.06	0.078
(Promoter [AePUb] × Context [Lep_P10]) × Rep [MA37]	0.35	0.21 – 0.60	**<0.001**
(Promoter [OpIE2] × Context [Kozak_SV40]) × Cell Line [U4]	1.05	0.64 – 1.73	0.856
(Promoter [shortHsp83] × Context [Kozak_SV40]) × Cell Line [U4]	1.74	1.06 – 2.86	**0.030**
(Promoter [Hsp83] × Context [Kozak_SV40]) × Cell Line [U4]	1.02	0.62 – 1.67	0.951
(Promoter [CqPUb] × Context [Kozak_SV40]) × Cell Line [U4]	0.49	0.29 – 0.84	**0.010**
(Promoter [400AePUb] × Context [Kozak_SV40]) × Cell Line [U4]	1.27	0.77 – 2.09	0.351

(Promoter [800AePUb] × Context [Kozak_SV40]) × Cell Line [U4]	1.37	0.83 – 2.25	0.222
(Promoter [AePUb] × Context [Kozak_SV40]) × Cell Line [U4]	0.95	0.57 – 1.56	0.828
(Promoter [OpIE2] × Context [Lep_P10]) × Cell Line [U4]	0.94	0.55 – 1.60	0.816
(Promoter [shortHsp83] × Context [Lep_P10]) × Cell Line [U4]	3.19	1.87 – 5.43	**<0.001**
(Promoter [Hsp83] × Context [Lep_P10]) × Cell Line [U4]	2.52	1.48 – 4.29	**0.001**
(Promoter [CqPUb] × Context [Lep_P10]) × Cell Line [U4]	1.68	0.95 – 2.97	0.076
(Promoter [400AePUb] × Context [Lep_P10]) × Cell Line [U4]	2.67	1.56 – 4.55	**<0.001**
(Promoter [800AePUb] × Context [Lep_P10]) × Cell Line [U4]	6.72	3.94 – 11.46	**<0.001**
(Promoter [AePUb] × Context [Lep_P10]) × Cell Line [U4]	4.10	2.40 – 6.98	**<0.001**
(Promoter [OpIE2] × Rep [MA34]) × Cell Line [U4]	0.47	0.28 – 0.81	**0.006**
(Promoter [shortHsp83] × Rep [MA34]) × Cell Line [U4]	2.58	1.51 – 4.40	**0.001**
(Promoter [Hsp83] × Rep [MA34]) × Cell Line [U4]	2.01	1.18 – 3.42	**0.010**
(Promoter [CqPUb] × Rep [MA34]) × Cell Line [U4]	0.81	0.46 – 1.44	0.478
(Promoter [400AePUb] × Rep [MA34]) × Cell Line [U4]	0.71	0.41 – 1.21	0.203

(Promoter [800AePUb] × Rep [MA34]) × Cell Line [U4]	1.53	0.90 – 2.61	0.117
(Promoter [AePUb] × Rep [MA34]) × Cell Line [U4]	0.56	0.33 – 0.96	**0.033**
(Promoter [OpIE2] × Rep [MA37]) × Cell Line [U4]	1.02	0.60 – 1.75	0.929
(Promoter [shortHsp83] × Rep [MA37]) × Cell Line [U4]	0.99	0.58 – 1.68	0.960
(Promoter [Hsp83] × Rep [MA37]) × Cell Line [U4]	0.85	0.50 – 1.45	0.543
(Promoter [CqPUb] × Rep [MA37]) × Cell Line [U4]	0.25	0.14 – 0.45	**<0.001**
(Promoter [400AePUb] × Rep [MA37]) × Cell Line [U4]	0.93	0.54 – 1.58	0.785
(Promoter [800AePUb] × Rep [MA37]) × Cell Line [U4]	1.42	0.83 – 2.42	0.196
(Promoter [AePUb] × Rep [MA37]) × Cell Line [U4]	0.87	0.51 – 1.48	0.601
(Context [Kozak_SV40] × Rep [MA34]) × Cell Line [U4]	1.47	0.92 – 2.33	0.104
(Context [Lep_P10] × Rep [MA34]) × Cell Line [U4]	1.21	0.71 – 2.06	0.487
(Context [Kozak_SV40] × Rep [MA37]) × Cell Line [U4]	1.03	0.65 – 1.64	0.888
(Context [Lep_P10] × Rep [MA37]) × Cell Line [U4]	0.52	0.31 – 0.89	**0.017**
(Promoter [OpIE2] × Context [Kozak_SV40] × Rep [MA34]) × Cell Line [U4]	1.36	0.67 – 2.75	0.394
(Promoter [shortHsp83] × Context [Kozak_SV40] ×	0.77	0.38 – 1.55	0.457

Rep [MA34]) × Cell Line [U4]			
(Promoter [Hsp83] × Context [Kozak_SV40] × Rep [MA34]) × Cell Line [U4]	1.08	0.54 – 2.20	0.822
(Promoter [CqPUb] × Context [Kozak_SV40] × Rep [MA34]) × Cell Line [U4]	1.23	0.59 – 2.57	0.576
(Promoter [400AePUb] × Context [Kozak_SV40] × Rep [MA34]) × Cell Line [U4]	1.36	0.67 – 2.75	0.395
(Promoter [800AePUb] × Context [Kozak_SV40] × Rep [MA34]) × Cell Line [U4]	0.49	0.24 – 1.00	0.051
(Promoter [AePUb] × Context [Kozak_SV40] × Rep [MA34]) × Cell Line [U4]	1.22	0.60 – 2.46	0.586
(Promoter [OpIE2] × Context [Lep_P10] × Rep [MA34]) × Cell Line [U4]	1.51	0.71 – 3.21	0.284
(Promoter [shortHsp83] × Context [Lep_P10] × Rep [MA34]) × Cell Line [U4]	0.58	0.27 – 1.22	0.151
(Promoter [Hsp83] × Context [Lep_P10] × Rep [MA34]) × Cell Line [U4]	0.74	0.35 – 1.57	0.433
(Promoter [CqPUb] × Context [Lep_P10] × Rep [MA34]) × Cell Line [U4]	1.26	0.58 – 2.77	0.557
(Promoter [400AePUb] × Context [Lep_P10] × Rep [MA34]) × Cell Line [U4]	1.07	0.50 – 2.28	0.857
(Promoter [800AePUb] × Context [Lep_P10] × Rep [MA34]) × Cell Line [U4]	0.37	0.17 – 0.78	**0.009**

(Promoter [AePUb] × Context [Lep_P10] × Rep [MA34]) × Cell Line [U4]	1.45	0.68 – 3.08	0.334
(Promoter [OpIE2] × Context [Kozak_SV40] × Rep [MA37]) × Cell Line [U4]	0.85	0.42 – 1.72	0.647
(Promoter [shortHsp83] × Context [Kozak_SV40] × Rep [MA37]) × Cell Line [U4]	0.82	0.41 – 1.67	0.587
(Promoter [Hsp83] × Context [Kozak_SV40] × Rep [MA37]) × Cell Line [U4]	1.41	0.70 – 2.86	0.336
(Promoter [CqPUb] × Context [Kozak_SV40] × Rep [MA37]) × Cell Line [U4]	3.27	1.57 – 6.80	**0.002**
(Promoter [400AePUb] × Context [Kozak_SV40] × Rep [MA37]) × Cell Line [U4]	1.08	0.53 – 2.18	0.838
(Promoter [800AePUb] × Context [Kozak_SV40] × Rep [MA37]) × Cell Line [U4]	0.96	0.48 – 1.95	0.919
(Promoter [AePUb] × Context [Kozak_SV40] × Rep [MA37]) × Cell Line [U4]	2.71	1.34 – 5.49	**0.006**
(Promoter [OpIE2] × Context [Lep_P10] × Rep [MA37]) × Cell Line [U4]	1.29	0.61 – 2.74	0.512
(Promoter [shortHsp83] × Context [Lep_P10] × Rep [MA37]) × Cell Line [U4]	1.37	0.64 – 2.92	0.412
(Promoter [Hsp83] × Context [Lep_P10] × Rep [MA37]) × Cell Line [U4]	1.70	0.80 – 3.61	0.170

(Promoter [CqPUb] × Context [Lep_P10] × Rep [MA37]) × Cell Line [U4]	3.17	1.45 – 6.91	**0.004**
(Promoter [400AePUb] × Context [Lep_P10] × Rep [MA37]) × Cell Line [U4]	1.59	0.75 – 3.38	0.228
(Promoter [800AePUb] × Context [Lep_P10] × Rep [MA37]) × Cell Line [U4]	0.81	0.38 – 1.72	0.584
(Promoter [AePUb] × Context [Lep_P10] × Rep [MA37]) × Cell Line [U4]	2.86	1.34 – 6.07	**0.006**
Observations	1218		
R^2 Nagelkerke	0.999		

Table S5. ANOVA table F values calculated from stepwise removal of coefficients from full model (Table S4).

Term	Df	F value	Pr(>F)
Promoter	8	354.91	< 0.001
Context	2	509.96	< 0.001
Residuals	1207		
Promoter: Context	14	7.255	< 0.001
Residuals	1193		

4. Methods

All cell lines were maintained at 28˚C, without CO_2 or humidity control. Aag2, U4.4 and C6/36 cells were maintained in L-15 (Thermo Fisher Scientific, Waltham, MA, US) supplemented with 10% FBS (Labtech, Lewes, UK), 1%Pen/Strep (Thermo Fisher Scientific, Waltham, MA, US) and 10% Tryptose Phosphate Broth (Thermo Fisher Scientific, Waltham, MA, US). Hsu cells were maintained in Schneider's Drosophila Medium (Lonza, Basel, Switzerland) supplemented with 10% FBS (Labtech, Lewes, UK) and 1% Pen/Strep (Thermo Fisher Scientific, Waltham, MA, US). Sf9 cells were maintained in Insect Xpress (Lonza, Basel, Switzerland) supplemented with 10% FBS (Labtech, Lewes, UK) and 1% Pen/Strep (Thermo Fisher Scientific, Waltham, MA, US).

Table S6. Transfection amounts per cell line	Aag2	C6.36	Hsu	Sf9	U4.4
FF plasmid (ng/well)	1	1	1	5	1
RL plasmid (ng/well)	50	5	5	1	5
TransIT Pro reagent (µl/well)	0.2	0.2	0.2	0.2	0.2
TransIT Boost reagent (µl/well)	0.1	0.1	0.1	0.1	0.1

4.1 Dual luciferase assay

Within an experiment, different cell lysate volumes (usually two) were screened for each cell line; and were used to select a lysate volume for the rest of the samples in that cell line. Each single luciferase control is represented, as is a double luciferase sample that is expected to have high expression of firefly luciferase (FF). The same two repeats are used at each lysate volume, but the FF plasmid in the double positive sample ("FF and RL") is not the same as that in the single positive ("FF only") sample.

In Figure S1 the FF and RL measurements of each sample are indicated as different colours with the transfection condition (combination of plasmids) represented on the x-axis and arbitrary light units (ALU) on the y-axis. The two lysate volumes, 1µl and 4µl are represented on independent graphs with the cell line indicated above (Aag2, in this case). Although the specific FF quenching threshold (106

ALU) is indicated, an estimated RL background threshold must be used as there is not enough data to calculate the 99.9% confidence interval it is based upon.

In optimisation experiment 2 (Figure S1), both lysate volumes show the desired pattern of results: each luciferase activity present above background threshold only where the corresponding luciferase plasmid was present in the transfection and FF activity present below the FF quenching threshold (106 ALU). It was decided to proceed with 1µl lysate volume for the rest of the experiment as RL measurements are higher without corresponding FF measurements becoming close to the quenching threshold (there may be experimental samples with greater FF activity than in the samples screened here). The volumes used are given in Table S7.

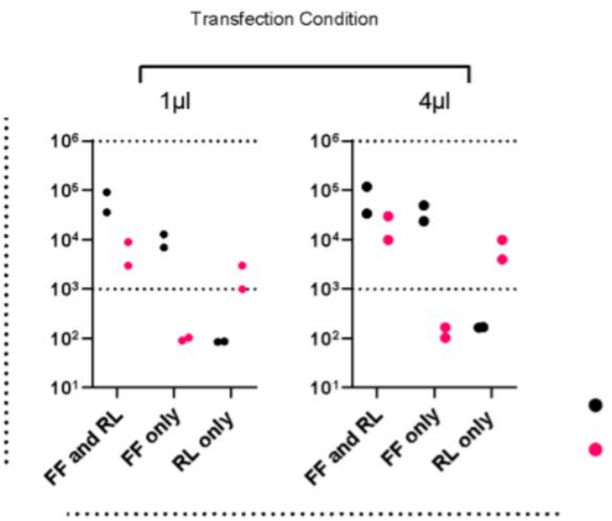

Figure S1. Indicative lysate volume. Graphs of lysate controls for optimisation experiments. Samples of control transfections were processed by dual luciferase assay at two volumes (1 and 4 µl). Each represented on a separate panel, with Arbitrary Light Units (ALU) on the y-axis.

Table S7. Indicative lysate volume for experiments

	Lysate volume (µl)				
	Aag2	C6.36	Hsu	Sf9	U4.4
Optimisation experiment 1	1	-	-	-	1
Optimisation experiment 2	4	-	-	-	-

Experiment	1	1	1	1	7

5. DNA sequences

Table S8. DNA sequences used in this project.

Name	Sequence
Kozak (Koz)	GCCGCCACC**ATG**G
Lepidopteran (Lep)	AACCAACAAC**ATG**G
B. mori High (BmHi)	AAAAATCAAA**ATG**G
B. mori Low (BmLo)	CCGCCGGCGT**ATG**G
Syn21 (Syn21)	AACTTAAAAAAAAAAATCAAA**ATG**G
Hr5-IE1 sequence	GCTTTACGAGTAGAATTCTACGCGTAAAACACAATCAAGTATGAGTCATAATCTG<br>ATGTCATGTTTTGTACACGGCTCATAACCGAACTGGCTTTACGAGTAGAATTCTA<br>CTTGTAATGCACGATCAGTGGATGATGTCATTTGTTTTTCAAATCGAGATGATGT<br>CATGTTTTGCACACGGCTCATAAACTCGCTTTACGAGTAGAATTCTACGTGTAAC<br>GCACGATCGATTGATGAGTCATTTGTTTTGCAATATGATATCATACAATATGACTC<br>ATTTGTTTTTCAAAACCGAACTTGATTTACGGGTAGAATTCTACTTGTAAAGCACA<br>ATCAAAAAGATGATGTCATTTGTTTTTCAAAACTGAACTCGCTTTACGAGTAGAA<br>TTCTACGTGTAAAACACAATCAAGAAATGATGTCATTTGTTATAAAAATAAAAGC<br>TGATGTCATGTTTTGCACATGGCTCATAACTAAACTCGCTTTACGGGTAGAATTCT<br>ACGCGTAAAACATGATTGATAATTAAATAATTCATTTGCAAGCTATACGTTAAAT<br>CAAACGGACGCTCGAGGTTGCACAACACTATTATCGATTTGCAGTTCGGGACATA<br>AATGTTTAAATATATCGATGTCTTTGTGATGCGCGCGACATTTTTGTAGGTTATTG<br>ATAAAATGAACGGATACGTTGCCCGACATTATCATTAAATCCTTGGCGTAGAATT<br>TGTCGGGTCCATTGTCCGTGTGCGCTAGTAGCATGCCCGTAACGGACCTCGTACT<br>TTTGGCTTCAAAGGTTTTGCGCACAGACAAAATGTGCCACACTTGCAGCTCTGCA<br>TGTGTGCGCGTTACCACAAATCCCAACGGCGCAGTGTACTTGTTGTATGCAAATA

	AATCTCGATAAAGGCGCGGCGCGCGAATGCAGCTGATCACGTACGCTCCTCGTG<br>TTCCGTTCAAGGACGGTGTTATCGACCTCAGATTAATGTTTATCGGCCGACTGTTT<br>TCGTATCCGCTCACCAAACGCGTTTTTGCATTAACATTGTATGTCGGCGGATGTTC<br>TATATCTAATTTGAATAAATAAACGATAACCGCGTTGGTTTTAGAGGGCATAATA<br>AAAGAAATATTGTTATCGTGTTCGCCATTAGGGCAGTATAAATTGACGTTCATGT<br>TGGATATTGTTTCAGTTGCAAGTTGACACTGGCGGCGACAAGCAATTGGTACCC<br>GGGTAGGATCCTAGTGAATTCCTAATCTGGCGGTAAGTTGATCAAAGGAAACGC<br>AAAGTTTTCAAGAAAAAACAAAACTAATTTGATTTATAACACCTTTAGAAGCC
AePUb	CCGCGGTATCTTTACATGTAGCTTGTGCATTGAATCCAATTATAATTTGCCTTGGC<br>ACCAGCTGAGCCAGACAAGAAAGAAAGCTTCCCAGAAGTATATCGATTTAGAAG<br>GGTTGACGTCACTTGCTGACTGCACTAATACAGCAAATGATGCAATTAGAATGAT<br>TCAAGTGAAATTCCCAAATTACTGATTTTTCTCTGGATTTGGTTATCAGATTACAT<br>TCGAAGCTAAGATTAGCTACCGAAATTGTCGATCAAATCAGGAAATCCTTTCTCT<br>ATCGAAAAAGGCATTCGCACATCTTCCTCTCTATGCCATATACACGAAGGGTAGG<br>TACATTGACGTCTTTGCCAGAAGTTGAACTGCATCGTTCAAGGTACAGAATGAAC<br>GACTAACAGACACAAGCACGTTTTGCTGTCCATTCAGACACAGGGATGGTACCC<br>ATATTCGATCGATATAGAGCCATCCAACCGAACAGAGGTATATGTATGAATGTAT<br>TGCTGAAATTTTCTAGAAGTACAACCACCACTACGACAGTGTCTATAAAACGCCC<br>CTGCAAAGGCGAAACCAGCTCAATCGAATACGTTTCCTAGTGGAGTGAACATTA<br>CGCGGCCCAAGTAAGCAGTGCCAGTGCAAGTGAAGTGAAGTCTCTAGTGAAAA<br>AGAGTGATCCAATTAGCCAGAGGAGAAAATTTCAGAGTGAACAAAGCTTTATTC<br>AAAGGACAATTACTATTAAATTGGTGAAAGTGCATTTCGGTGAAGGGAATCTTCT<br>AGTGAAGGTAGGTAAATTAATTGATGAAATTATAGCTATGAGCGAAAACTAGTT<br>TGGTGAATGATTCCTTTGTCTTTGAATGAGCAAACTATTTTCCAAGATGGCGACT<br>ATTGAGCTTTGAGTGATTAGTGAAAATTTGCAACGCAGTTTCATCATCATTGATA<br>AAACCCAATTGTGATTCACAGCGATAATCATATTTCGTTGAATCATCGCTACTAAT<br>TGAATTAAATTTCTAGAATAATAAGAATAACGTATTTGCTCCGTCACATATCTAAA<br>ATAAATATTTTGATGGTAATTACCCATTAAGGTAATATTAACACATATCGAGAAA<br>AACCTTGAGGAAATCGTGAAAACTTGAAGATACGCAATTTCAAAACTACGTAGTT<br>CAAAGTCGAAAACAAGTTAATTTTTCACTTAAAAGTAGGGCGTTGTTGTGACGTC<br>ATCACCTTCAAGTGTATATTTTTCACTTGGCCTGCGACTGCAAACGCAGACAAAG<br>CAAAACAAGTTTAAAACCTGTCGTGTCGTGCTCGAAGCCAAAGGCAATGAATCA

	ATATCAAATGAGAGTTTGCATTTCACAACCAATTACTCAAGCGTTTCCTCGTTTCT TTTTCTGCTCAACAGAGATTTCC
Alb PUb	TGCCCTGTCCGACTACAATGTTCAGAAGGGATCTACCCTGCACTTGGTGCTACGC CTCCGAGGTGGTATGCAGATCTTCGTCAAGATGTTAACTGGCAGGTGCATGGCC ATCGATACCGAACCAGAGGACACAGTCGACACATTGAGGGTTAAGATCTCGGAG AAACTGGAAGAAATGCCACCAAACCAGCTACGTCTGATCTTCGCCGGTAAGCAA TTGGAGGATGGACGCACCTTACAGGAATACAGCATCATCAAGGGTTCGACAGTC CATCTGGTTCTGAGGTTAAAGGGCGGTCAATGACGAACTATGCGAAGAACCCCG ACTGGAAACTGCAGGAATACAGCATCATCAAGGGTTCGACAGTCCATCTGGTTCT GAGGTTAAAGGGCGGTCAATGACGAACTATGCGAAGAACCCCGACTGGAAACT GGAACTACATGGAACTGCCTCCAAGACTTGTACGCGAAGCAAAGTGAATCTTTG CGAAGAAACTACCAAATGAAACAATTTATAATTTAGGAAATTAGCTAGCTAAACT CTGTGCATACATTAAATAAAATCGAACCACTCGAAGTGATATCTTTACATGTAGC TCGCCTGTGCATTGAATCCAATTATAGTTTGCCTTGGCACCAGCTGAGCCAGACA AGAAAGAAAGTGCTTCCCTGAAGTATATTGATTTTAGAAGGCCTGACGTCAGCA GATGGCTGCACTAATACAACAAGTGATGCAATTAGAATGATTCAAATGAAATTCC CAAATTACTGGTTTTCTTATAGAATTCAGTTATCATATTACATTGAAAGTCTAAAT TTGTTACCTAAACTATTGGTAAATTCAGCAAACTCTTTCTCATCTTTCATGAAAGG CATTCGCATATCTTCCGACAGCATATATGCGACGGTAGGTACATTGACGTCTTTG CCAGAAGTTAAACCACATCGTTCAAGGTAGAAAAATACAGAATGAACCAACAAA TACAAGCACCATTCAGTTCGACAAACAGAAGAGGGTAGTGAATCGATACGGTCA ACCGCATAGATATACCTAGATGTGTATTGCTACAATTTTCTAGAAAtACAACTACC ACGaCAGTAGTTGGCTATAAAGTCTCCgTGCAAAAGCGAACCCAGCTCAATCGAA TACGTTTCCTAGTGGAGTGAACACACCAAAACGGTCCAAGTAGGCAGTGCACCA GTGCAAGTGAAGTGAAAAAGACAAGAGTAATCCAATTAGCCAAGCGGAGAAAA TTCCAGAGTGAACGAAcATTTGAAAAAAGGACTTTGCTCGAAGCGAGTTTAAATT TGTGAAAGTGCATTTCGGTGAAGGAATCTTCTTGAAAAGGTAAGGGGGTGATTG GATCGATTAGAATAGATGTGAATAAGTGAAAACTAGTTTGTGGGTGATTTCTTTG TCTTTGAATGAGCAAATATATTTTTCAAGATGGCGACTGTAATTGATGAAAAATT TGCAGCGAAGTTTCATCATCATTGATAAAACCAAGATGTGATTCACAACGATAAT AATATTTTATTGAATCATCGACGTTAGTCGAAAGAATAATCTGGAATATAAGAAG CCCTCCATTATTAATTTGAACGCTTAAGTGATGGGTTCTTATAACTTATTAAGGTA ATACTGACGTTCATAACGAAGGGCCTTGAGGGTATATTTAAAACTTTTAGAGATA

	CGCAATTTCCCAAGTCTCAACTACGTAGTTAAAAGTGAAAAACAACCTGATTTTC CAATTAAAAGTAGGGCGTTGTTGTGACGTCATCACCTGCACGTAGATTTTTCACA TCGTCTGCGACTGCGAATGCGAACGCAAGACAAAGCAAAATAAAATCGAAACCT GTCTTGTCGTGCTCAAACCACAAAGCAATGAATCAACATAAAGTGAGTTTGTgTT TCGCAATCATTACTAACGTACTTGTTTCCTTGTTTCTATTTCTGGTTCAACAGAGAT TTCAACC
AeHsp83	CAATTCTACTGCACTGAAATGACCTGTCTGGCAAACTGTATGGTGGACTGTATAA AACTGAATACCGGAAACACTCTTACATCGCTTTCAGATTGACATCTGTCATCTACA TCAAAATTGCAGCGAAGCTTTGACAGAAGGAAGCGTCGCATTAAAGGCGGAGG TTGGCCGGGTTGCCAGCTTGCCGCCTCCTGCGCCGACAAATTGTTTTGAACATTT TTTTTCAAATACCATTATAATACCAAAATGAGGTATTGACAACTGATCAATACCTA ATTATGGTATGATACCAAAATATGGTATGCATAAGTTATTGCGAGTTATTCTTTCC TCCTCGGGCAGAGTTGCCAGTTCAAATAGGACATATCGATACTAGAAAAGCTTTA TAAGAGAGATTCGCGGAAGCTGACATTTCTGTCAAAGTGTGAGCCAATCGAGCA GCGAGAGCTGTCAAAGTGTGAGCCAAACGAACATGCGTTATGTTTGTTTTGAACT TTTCTTGAGGAGTAATGTAATCCGCTTGAAATTATTTCGGTAAAAGTAATTTCGCA TGAAGCAAAACAAATGAAATGTTTTACTTTTTTGAATTTTTGTCAAAGCGATTTTT AGTTTTTGATTTTTTCGGCTGTTTATTAGTTTTGATATGTAGCTCAAAGGTCTATA ACGAAGCAAAACACACTTTATAGCAATGAGGAAGTCATGTCCGTGTTACAATATT ATTCTCGACGCCGAGCAAAATCAGCACTGCTGGTCTGTTGCCAAATCATTCCATTT TACTTCCGCTTATCTCTCTATAAAGGATCACTAATCGATACGGTCGCTCACTTTGG CACAGTCGGCACAAATCGGCACAATTTTAGGTGAGCCGAGTTTCCCTTCTTCTTCT TTGTGGCGTTACGTCCCCACTGGGACAGAGCCTGCTTCTCAGCTTAGTGTTCTTAT GAGCACTTCCACAGTTATTTACTGAGAGCTTACTATGCCAATGACCATTTTTGCAT GTGTATATCGTGTGCTTACTATGCCAATGACCATTTTTGCATGTGTATATCGTGTG GCAAGTACGAAGATACTCTGTGCCCTGGGAAGTCGAGAAAATGTCCAATCCGAA AAGATCCTCGACCGGTGGGATTCGAACCCACGACCCTCAGCTTGGTCATGCTTAA TAGCTGCGCGTTTATCACTACGGCTATCTGGGCCGAGTTTCCCACATCCTGCAAT AATCCATCCTCCAAATAAATCCACAATTTATTTATATTATATAATATCTTTGAACAA AATTGGCTCTATTACTTTTTCGACCATACAATCGAAACCCTCTACTAAGCGCTCTC TCTCACACTACTCAAATCTATCGTATCAGCTTTCACTCACACATCGCGATGCGATG CCGGTTCCGCAACCGTGTCGTGGAGGGGAAAATCGCGAATCGAATCGGTTAAGC CGAACAAAGCCGATCACACTTTTGCCAACCGGTTGTTTAAATCTCCCGCTCTCCGC

	TTCGATTCCGTCGATTCATTCTGTAAAAACCGGTTGACTGTTCCAGACGCATTTGC<br>GCTCGGTGTTGTAAGAAGTGGTTCGAGAAAAAGTGCCCACCAGAAAATTCCCGA<br>ATTTTCTCCGGAAGAAAAAAACGGGTCTTCTTCTAGAAAGAGTGTGATCAGAGG<br>AGGAAAAACCGTCGGTGATTTTTGTGTTGTGTTTCTAGTGATCCGGAAGGACAA<br>CAAACGCTCAAAGTCAAGGTGAGTGCCGTCGATAAATTTCATTGTCCGGGAGGA<br>GTCGTAGCGGCTTTTACACAGGGGGAACAAGCCGCTGCGCAAAATCTGGAAAGC<br>ACGTTTTGCTTCGCTGAATTCGAGGCCGATCTTTTGTAATTTACCGTTGATTTAGT<br>GAATCTTGTAATCTGTTATATATCACATTGCAGATCAGCTTGCTTGTAGTAGACAA<br>AGCACGTGTTTTCCTCCAATTTATCCCTTTTCACCATTAACTATTAAATACGAATTT<br>AGTCTAAGCGTCTTGTTTTTCCTTCTTCACTCCGTTCCAGATCCCGGAAGCC
OPIE-2	CATGATGATAAACAATGTATGGTGCTAATGTTGCTTCAACAACAATTCTGTTGAA<br>CTGTGTTTTCATGTTTGCCAACAAGCACCTTTATACTCGGTGGCCTCCCCACCACC<br>AACTTTTTTGCACTGCAAAAAAACACGCTTTTGCACGCGGGCCCATACATAGTAC<br>AAACTCTACGTTTCGTAGACTATTTTACATAAATAGTCTACACCGTTGTATACGCT<br>CCAAATACACTACCACACATTGAACCTTTTTGCAGTGCAAAAAAGTACGTGTCGG<br>CAGTCACGTAGGCCGGCCTTATCGGGTCGCGTCCTGTCACGTACGAATCACATTA<br>TCGGACCGGACGAGTGTTGTCTTATCGTGACAGGACGCCAGCTTCCTGTGTTGCT<br>AACCGCAGCCGGACGCAACTCCTTATCGGAACAGGACGCGCCTCCATATCAGCC<br>GCGCGTTATCTCATGCGCGTGACCGGACACGAGGCGCCCGTCCCGCTTATCGCG<br>CCTATAAATACAGCCCGCAACGATCTGGTAAACACAGTTGAACAGCATCTGTTCC<br>TGCAGAAGTTGGTCGTGAGGCACTGGGCAGGTAAGTATCAAGGTTACAAGACA<br>GGTTTAAGGAGACCAATAGAAACTGGGCTTGTCGAGACAGAGAAGACTCTTGCG<br>TTTCTGATAGGCACCTATTGGTCTTACTGACATCCACTTTGCCTTTCTCTCCACAG<br>GTGTCCACTCCCAGTTCAATTACAGCTCTTAAGGCTAGAGTACTTAATACGACTCA<br>CTATAGGCTA
CQ PUB	TTATTCTTTAAAGTTATACTCGATTGAGTGTTTGGTATGCGTCAAAAAAATCTGTA<br>TTTGTTGCAAGCCTGCTCACAAACTACCAAACGTTTGTTTTGATAGTGTGCGTGA<br>GCGCCGTGTAAAAAGTGACAGTTCGTCATATTTTGTTTGACTTTGACCAACCTAC<br>GGGGTACAAACTAAAAAAGTGTCAAACGAAAAAGTGACCAACCACCGGGGGTT<br>GAGGGTACACTCAGAAAAAAGTACTGGCAAAATCCATAAGAACGACTTATGACC<br>TTTCGACATCAGGATTTTATTCGGATGTCATAAGATTTTCTTATGGCTGGGAGGG<br>GAAATTTGACATAGCCGTCAATTAAGATTTCCATTGAGTTATCTTATGACATTCAT

	GGATGGAATTGTGTACAGAATATATGGCAATGCTCATGGAGATTATATTAATAA<br>ACATTGTTTTTCATACCCATTTTTTCTTATTTTTAGTTAAATGAAAACAAAAATTTA<br>TTTAAATTTTAAACGTATATATTTTTAAATTATCGTAATATACCCAAAACTTCATTT<br>CCTTCCGCTGCTCGTCGTCCCGTTGGTTTACGCAGCCTCCGGTTTAATCGTCTTTT<br>GCTGGTAATTTGGCATGTTCTGCCGGATGGCCACCACCGCGTCAATCAATCTTAA<br>ACCTTCGACCGCTGCTCGGAACAGTCCTCACCGATGCTATTCTCCGGATCCTTCTG<br>TAGGATGCTGGGGAGGGCCGCCGGCGGAACCAACGTCCATTGATATTACCTAAA<br>ATACCAAAAACAAAATATTCAACTTCGAACCAAAACCCACGAAACACCCGCCACA<br>GAACTTACCTGTGTCTTGAAGGTCTCGCTAAAACTCATAGCGGCGGCGGCAGCTT<br>TGTGAAGTAGTTACCGGAACGAACCACGGGGCGATTTGGAGTAGCGAGCGTTTA<br>AAATTGTCTGCTCGATCCGTCCTTTCGCCATCTTGAAATGTCAAAAAATTTCACAC<br>TAGCATCATACGCGAAAACCATTGAAAAATTACAGATGAATTCCATAAGAATTTC<br>TTATGAAGTCCATAGTCGATCAACAGTGGACAAAATACATAAGTAAAGATTAGTT<br>ATAACCATATGAATTTATAGTGACGGTCATAAGATTGTCTATGAAAATCGCGAGA<br>GCCTAGTGGTTACTCAAATCAGGCAGCACATAAGATGTAGTCTTATGAAAATCTT<br>ACATTCTTTTTCCTCAGTGTACATAAAATCTTTATTTTTATTAGCTAAATTAGTTTT<br>ATAGCAACTGTCTTGTCGTCCTAATCGACAAATCCCTCAATCAACTCGCAATTTTA<br>TAGCACTGCAACAATCCCAGAGGGAGTCAGCAGCCGCCGGAGAGCAGCTGTCA<br>AACCGCGAACCGAGCGTCATCGTCGCAGAGGGAAGAACCAGATAAAGTTCCGCT<br>AACACCTTGAAGTTTTGCAGAGGAAAAATTCGATTTTCATCCGCATCGCACGAGC<br>CAAT
SV40	CAGACATGATAAGATACATTGATGAGTTTGGACAAACCACAACTAGAATGCAGT<br>GAAAAAAATGCTTTATTTGTGAAATTTGTGATGCTATTGCTTTATTTGTAACCATT<br>ATAAGCTGCAATAAACAAGTTAACAACAACAATTGCATTCATTTTATGTTTCAGGT<br>TCAGGGGGAGGTGTGGGAGGTTTTTTAAAGCAAGTAAAACCTCTACAAATGTGG<br>TA
K10	GGAGCTTGATAACATTATACCTAAACCCATGGTCAAGAGTAAACATTTCTGCCTT<br>TGAAGTTGAGAACACAATTAAGCATCCCCTGGTTAAACCTGACATTCATACTTGT<br>TAATAGCGCCATAAACATAGCACCAATTTCGAAGAAATCAGTTAAAAGCAATTAG<br>CAATTAGCAATTAGCAATAACTCTGCTGACTTCAAAACGAGAAGAGTTGCAAGTA<br>TTTGTAAGGCACAGTTTATAGACCACCGACGGCTCATTAGGGCTCGTCATGTAAC<br>TAAGCGCGGTGAAACCCAATTGAACATATAGTGGAATTATTATTATCAATGGGG

	AAGATTTAACCCTCAGGTAGCAAAGTAATTTAATTGCAAATAGAGAGTCCTAAGA<br>CTAAATAATATATTTAAAAATCTGGCCCTTTGACCTTGCTTGTCAGGTGCATTTGG<br>GTTCAATCGTAAGTTGCTTCTATATAAACACTTTCCCCATCCCCGCAATAATGAAG<br>AATACCGCAGAATAAAGAGAGATTTGCAACAAAAAATAAAGGCATTGCGAAAAC<br>TTTTTATGGGGGATCATTACACTCGGGCCTACGGTTACAATTCCCAGCCACTTAA<br>GCGACAAGTTTGGCCAACAATCCATCTAATAGCTAATAGCGCAATCACTGGTAAT<br>CGCAAGAGTATATAGGCAATAGAACCCATGGATTTGACCAAAGGTAACCGAGAC<br>AATGGAGAAGCAAGAGGATTTCAAACTGAACACCCACAGTACTGTGTACTACCA<br>CTGGCGCGTTTGGG
P10	ATGAATCGTTTTTAAAATAACAAATCAATTGTTTTATAATATTCGTACGATTCTTTG<br>ATTATGTAATAAAATGTGATCATTAGGAAGATTACGAAAAATATAAAAAATATGA<br>GTTCTGTGTGTATAACAAATGCTGTAAACGCCACAATTGTGTTTGTTGCAAATAA<br>ACCCATGATTATTTGATTAAAATTGTTGTTTTCTTTGTTCATAGACAATAGTGTGTT<br>TTGCCTAAACGTGTACTGCATAAACTCCATGCGAGTGTATAGCGAGCTAGTGGCT<br>AACGCTTGCCCCACCAAAGTAGATTCGTCAAAATCCTCAATTTCATCACCCTCCTC<br>CAAGTTTAACATTTGGCCGTCGGAATTAACTTCTAAAGATGCCACATAATCTAAT<br>AAATGAAATAGAGATTCAAACGTGGCGTCATCGTCCGTTTCGACCATTTCCGAAA<br>AGAACTCGGGCATAAACTCTATGATTTCTCTGGACGTGGTGTTGTCGAAACTCTC<br>AAAGTACGCAGTCAGGAACGTGCGCGACATGTCGTCGGGAAACTCGCGCGGAA<br>ACATGTTGTTGTAACCGAACGGGTCCCATAGCGCCAAAACCAAATCTGCCAGCGT<br>CAATAGAATGAGCACGATGCCGACAATGGAGCTGGCTTGGATAGCGATTCGAGT<br>TAAC

Table S9. Brief descriptors and Lab plasmid registry names. All plasmids registered with Genbank PP916442-PP916472.

Name	AGG number
HR5-IE1-Koz-FF-SV40	AGG1186
HR5-IE1-Lep-FF-SV40	AGG1187
HR5-IE1-BmHi-FF-SV40	AGG1188
HR5-IE1-BmLo-FF-SV40	AGG1189
HR5-IE1-Koz-FF-K10	AGG1191

HR5-IE1-Lep-FF-K10	AGG1192
HR5-IE1-BmHi-FF-K10	AGG1193
HR5-IE1-BmLo-FF-K10	AGG1194
HR5-IE1-Koz-FF-P10	AGG1196
HR5-IE1-Lep-FF-P10	AGG1197
HR5-IE1-BmHi-FF-P10	AGG1198
HR5-IE1-BmLo-FF-P10	AGG1199
AePUB-Kozak-FF-SV40	AGG1330
AePUB-Lep-FF-P10	AGG1331
AePUB-BmLo-FF-K10	AGG1332
400AePUB-Kozak-FF-SV40	AGG1333
400AePUB-Lep-FF-P10	AGG1334
400AePUB-B.mori_low-FF-K10	AGG1335
800AePUB-Kozak-FF-SV40	AGG1336
800AePUB-Lep-FF-P10	AGG1337
800AePUB-BmLo-FF-K10	AGG1338
AlbPUb-Kozak-FF-SV40	AGG1345
Hsp83-Kozak-FF-SV40	AGG1390
Hsp83-Lep-FF-P10	AGG1391
Hsp83-BmLo-FF-K10	AGG1392
sHsp83-Kozak-FF-SV40	AGG1393
sHsp83-Lep-FF-P10	AGG1394
shortthsp83-BmLo-FF-K10	AGG1395
OPIE2-Kozak-FF-SV40	AGG1396
OPIE2-Lep-FF-P10	AGG1397
OPIE2-BmLo-FF-K10	AGG1398
CQ PUb - Kozak - SV40	AGG1416
CQ PUb- Lep-FF - P10	AGG1417
CQ PUb – BmLo-FF-K10	AGG1418

References

R Core Team (2023). _R: A Language and Environment for Statistical Computing_. R Foundation for Statistical Computing, Vienna, Austria. <https://www.R-project.org/>.

Lenth RV. emmeans: (2023) Estimated Marginal Means, aka Least-Squares Means [Internet]. Available from: https://github.com/rvlenth/emmeans
